# Homeostatic plasticity of eye movement performance in *Xenopus* tadpoles following prolonged visual image motion stimulation

**DOI:** 10.1007/s00415-022-11311-8

**Published:** 2022-08-10

**Authors:** Michael Forsthofer, Hans Straka

**Affiliations:** 1 Faculty of Biology, Ludwig-Maximilians-University Munich, Großhaderner Str. 2, 82152 Planegg, Germany; 2 Graduate School of Systemic Neurosciences, Ludwig-Maximilians-University Munich, Großhaderner Str. 2, 82152 Planegg, Germany

**Keywords:** Optokinetic reflex, Cerebellum, Inferior olive, Ontogeny, Plasticity

## Abstract

Visual image motion-driven ocular motor behaviors such as the optokinetic reflex (OKR) provide sensory feedback for optimizing gaze stability during head/body motion. The performance of this visuo-motor reflex is subject to plastic alterations depending on requirements imposed by specific eco-physiological or developmental circumstances. While visuo-motor plasticity can be experimentally induced by various combinations of motion-related stimuli, the extent to which such evoked behavioral alterations contribute to the behavioral demands of an environment remains often obscure. Here, we used isolated preparations of *Xenopus laevis* tadpoles to assess the extent and ontogenetic dependency of visuo-motor plasticity during prolonged visual image motion. While a reliable attenuation of large OKR amplitudes can be induced already in young larvae, a robust response magnitude-dependent bidirectional plasticity is present only at older developmental stages. The possibility of older larvae to faithfully enhance small OKR amplitudes coincides with the developmental maturation of inferior olivary–Purkinje cell signal integration. This conclusion was supported by the loss of behavioral plasticity following transection of the climbing fiber pathway and by the immunohistochemical demonstration of a considerable volumetric extension of the Purkinje cell dendritic area between the two tested stages. The bidirectional behavioral alterations with different developmental onsets might functionally serve to standardize the motor output, comparable to the known differential adaptability of vestibulo-ocular reflexes in these animals. This homeostatic plasticity potentially equilibrates the working range of ocular motor behaviors during altered visuo-vestibular conditions or prolonged head/body motion to fine-tune resultant eye movements.

**Supplementary Information:**

The online version contains supplementary material available at 10.1007/s00415-022-11311-8.

## Introduction

Gaze-stabilizing eye movements ensure unblurred visual images during head and/or body movements. Such image-stabilizing eye movements appeared early during evolution in freely swimming aquatic vertebrate ancestors with subsequent phylogenetic improvement of the performance, likely related to the increasing complexity of locomotion, visual acuity, and motion repertoires [1–3]. Optimal execution of these reflexes requires real-time evaluation of the motor performance through sensory reafferences and computational predictions of visual motion to allow animals to adapt the output [4, 5]. While being the dominant contributor to gaze stabilization during head perturbations, the open-loop vestibulo-ocular reflex (VOR) is supplemented by closed-loop visuo-motor reflexes such as the optokinetic reflex (OKR) [6]. This latter ocular motor behavior is driven by residual large-field visual image motion that remains uncompensated for by the VOR, and provides the sensory feedback to optimize gaze stability [7]. The visuo-motor system thus enhances the adequacy of eye movements following integration of visual motion and vestibular signals, which occurs predominantly within cerebellar circuits [8]. In fact, the various afferent and efferent brainstem–cerebellar connections integrate multisensory motion signals [9, 10], but also form the neural substrates for implementing changes in sensitivity during active *versus* passive head movements [11] or during development and aging [8].

The role of the cerebellum in eye motion plasticity in mature vertebrates has been extensively studied using a variety of stimulus paradigms, species, and behavioral repertoires (e.g., [8]). The flexible interaction between vestibular and visual motion signals in cerebellar circuits has been the focus of numerous experimental studies (e.g., [12]; see [8]) largely based on earlier theoretical considerations [13, 14]. Key to the outcome of such studies was the importance of the connectivity between the inferior olive and the cerebellum in mediating adaptive plasticity [10, 15]. The majority of these studies explored the role of the cerebellum in adapting vestibular motion-evoked eye movements under specific sensory conditions (e.g., visuo-vestibular mismatch; [16]) or after a loss of head/body motion-related sensory signals (e.g., labyrinthectomy; [17]). In contrast, only few studies have so far evaluated the consequence of prolonged visuo-vestibular or pure visual motion stimulation and the potential role of the cerebellum in modifying ocular motor outputs (e.g., [15, 16, 18, 19]). Habituation of the VOR performance has been demonstrated during prolonged vestibular stimulation [20–22]. Conversely, improvement of VOR performance is possible but dependent on specific stimulus paradigms [23]. Increases of the OKR amplitude have been shown during prolonged vestibular or optokinetic stimulation [18, 24, 25]. Bidirectional, cerebellum-dependent changes of the angular VOR have been reported in *Xenopus laevis* tadpoles during prolonged rotation and were interpreted as homeostatic plasticity, adjusting the ocular motor output to a preset magnitude [26]. However, the ontogenetic onset and applicability of such a homeostatic plasticity to other ocular motor behaviors is so far unknown.

Here, we tested the impact of prolonged visual motion stimulation on the performance of the OKR in *Xenopus* tadpoles at different developmental stages, before and after the presumed onset of cerebellar function. The employment of semi-intact preparations for eye motion recordings, surgical manipulations, tract tracing, and immunohistochemical analyses, allowed demonstrating the involvement of the cerebellum in specific aspects of homeostatic OKR plasticity, once cerebellar neuronal elements have become functional.

## Materials and methods

### Animals and experimental preparation

*Xenopus laevis* embryos were obtained from the in-house breeding facility at the Biomedical Center or the Biocenter Martinsried of the Ludwig-Maximilians-University Munich. After hatching, tadpoles were reared at a temperature of 17–18 °C with a 12/12-h light/dark cycle in dechlorinated water.

All experiments were performed on semi-intact in vitro preparations of 97 tadpoles in compliance with the "Principles of animal care", publication No. 86-23, revised 1985 of the National Institute of Health and were carried out in accordance with the ARRIVE guidelines and regulations. Permission for the experiments was granted by the legally responsible governmental body (Regierung von Oberbayern) under the license code ROB-55.2–2532.Vet_03-17–24. In addition, all experiments were performed in accordance with the relevant guidelines and regulations of the Ludwig-Maximilians-University Munich.

Semi-intact preparations were obtained following a procedure described previously [27–29]. Animals of either sex at larval stages 50–51 (young tadpoles) and 55–56 (old tadpoles, [30]), respectively, were deeply anesthetized in 0.05% 3-aminobenzoic acid ethyl ester methanesulfonate (MS-222; Pharmaq Ltd.) dissolved in ice-cold frog Ringer solution (75 mM NaCl, 25 mM NaHCO_3_, 2 mM CaCl_2_, 2 mM KCl, 0.1 mM MgCl_2_, and 11 mM glucose, pH 7.4). In a Ringer-filled, Sylgard-lined dish (Sylgard 184, Dow), larvae were decapitated under a binocular microscope (SZX16, Olympus) at the cervical spinal cord. Following removal of the lower jaws and visceral organs, the head was mechanically secured dorsal side-up onto the Sylgard-lined floor with *minutiae* pins (0.2 mm, Fine Science Tools). Subsequently, the skin of the head and the cartilaginous tissue of the skull were opened, the *choroid* plexus covering the fourth ventricle was removed and the forebrain disconnected. Both eyes, including all extraocular muscles, were left intact and remained connected to the brain via the optic nerve and the extraocular motor nerves, allowing presentation and sensory–motor processing of visual motion stimuli and the recording of eye movements (see below). Following the dissection, semi-intact preparations were transferred into fresh frog Ringer solution and allowed to recover for 3 h at 17 °C.

Disruption of the olivary–cerebellar pathway in semi-intact preparations was performed by a longitudinal midline transection in rhombomeres (*r*) 7 and 8, the hindbrain segmental level where the axons of cerebellum-projecting inferior olivary neurons cross the midline [31]. Following the initial 3-h recovery period and two cycles of stimulation to capture the reference OKR performance, the surgical intervention was performed under visual guidance using *minutiae* pins (0.1 mm). The target site for the transection was identified by external landmarks such as the lateral exit/entrance of cranial nerves IX–XI in r6 and r7 [32]. Thereafter, preparations were allowed to recover from the procedure for 30 min at 17 °C in darkness and were subsequently subjected to visual image motion stimulation as described below. The successful disruption of inferior olivary axons was histologically confirmed after each experiment (see below).

### Visual motion stimuli, optokinetic training, and eye motion recording

**Fig. 1:**
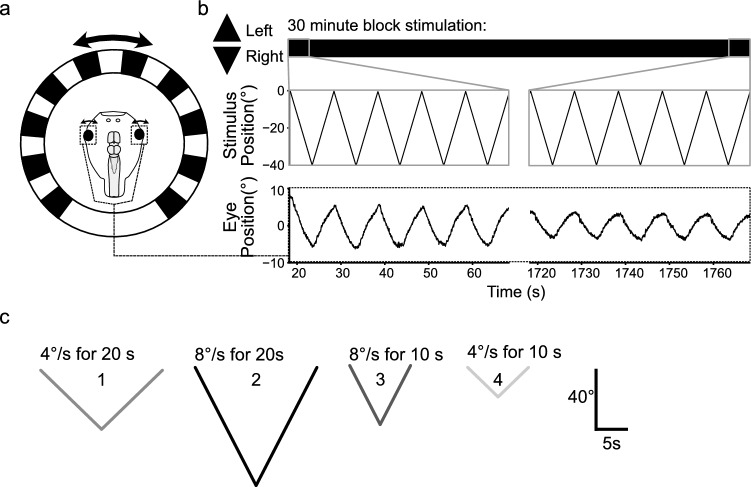
Stimulation and recording of the optokinetic reflex in semi-intact preparations of *Xenopus laevis* tadpoles.** a** Schematic illustrating the experimental setting with a Ringer-filled circular recording chamber hosting the preparation; horizontal motion of vertical black and white stripes across the surrounding cylindrical screen serves as large-field visual motion stimulus and elicits eye movements (double arrows). **b** Representative example of horizontal positional oscillations of the eyes (lower trace) during prolonged (30 min) visual motion stimulation (upper traces), at the onset (left) and the end of the training period (right). **c** Single cycles of visual image motion profiles depicting the different combinations of stimulus velocities of either 4°/s (1, 4) or 8°/s (2, 3), presented in bidirectional alternation with a cycle duration of either 20 s (1, 2) or 10 s (3, 4)

For eye motion recordings, semi-intact preparations were mechanically secured dorsal side-up in the center of a Sylgard-lined circular dish (Ø 5 cm), surrounded by a visual virtual reality environment as described previously (Fig. 1a; [29]). The visual environment consisted of equally spaced, vertical black and white stripes subtending a visual angle of 16° per stripe. This pattern was projected onto a cylindrical screen (275° coverage, Ø 8 cm, 5 cm height; Fig. 1a) at 60 Hz by three digital light processing video projectors (Aiptek V60), affixed in 90° angles to each other on the experimental table. For the optokinetic training, the visual image motion pattern was continuously oscillated horizontally to the left and right for 30 min (Fig. 1b). Stimulus motion consisted of alternating bidirectional movements at two velocities: 4°/s or 8°/s, presented at different stimulus cycle durations—for 10 s or for 20 s—to generate visual image motion with different peak-to-peak position amplitudes. Accordingly, these stimulus parameter combinations produced four different profiles (Fig. 1c), and correspondingly elicited OKR responses with different eye motion amplitudes. Each animal was tested for only one stimulus profile to avoid transferring a possible training effect from an initial training stimulus onto a subsequent one. Accordingly, each stimulus profile was separately tested on a group of animals naïve to any training stimulus.

The movement of both eyes was captured non-invasively from above with a camera (Grasshopper 0.3 MP Mono FireWire 1394b, PointGrey, Vancouver, BC, Canada) equipped with an adequate objective lens and infrared-Filter (800 nm long pass) at a frame rate of 30 frames per second, while the preparation was illuminated from above by an infrared light source (840 nm). Eye positions were extracted in real time from the video by fitting an ellipse around each eye [27]. The angle between the long axis of each ellipse and the horizontal image axis was calculated in a frame-by-frame manner by a custom-written software [29, 33] and was recorded and stored for off-line analysis along with the visual motion stimulus (Spike2 version 7.04, Cambridge Electronic Design Ltd.).

### Data analysis

Data acquired in Spike2 were exported in the MATLAB (The MathWorks Inc.) file format and analyzed off-line with custom-written Python scripts. Due to irregular sampling of stimulus and eye positions, stimulus and eye motion recordings were resampled at 200 Hz and filtered with a 4 Hz low-pass Butterworth filter. Traces were segmented into individual stimulus cycles from peak-to-peak, and conjugate motion traces of the left and right eye were averaged to generate a joint response of both eyes (see [28]). Infrequent cycles with either stimulus-evoked fast phases or spontaneous jerking movements were manually identified and excluded from further analysis. Following this preprocessing, peak-to-peak amplitudes of eye movements were determined for each motion cycle, to allow detection of amplitude changes across the training session. In addition, responses to the first and last five stimulus motion cycles of a given training period of 30 min were averaged, respectively, to generate a mean initial and a mean entrained response to directly compare the peak-to-peak motion amplitude of the original and entrained OKR response. Statistical analysis and plotting were performed in Prism 9 (GraphPad Software Inc.). Representative eye motion traces were normalized and averaged prior to plotting. Data, comparing responses of individual animals, were plotted as column scatter plots with or without mean ± SD. Statistical differences between experimental groups were calculated with the non-parametric Mann–Whitney *U*-test (unpaired parameters), the Wilcoxon signed-rank test (paired parameters), or the Kruskal–Wallis test and a Dunn’s test (unpaired parameters) for multiple comparisons and indicated as *p*-values (*p < 0.05; **p < 0.001; ***p < 0.0001).

### Neuronal tracing

The connectivity between inferior olivary neurons and the cerebellum was anatomically profiled after completion of the behavioral experiments by fluorescent labeling of the neuronal projections in control tadpoles and those which received an experimental midline transection (see above). Tetramethylrhodamine, conjugated to 3000 MW Dextran (Invitrogen, D3308), was dissolved in ddH_2_O, and crystallized onto 0.1 mm *minutiae* pins. Preparations were mechanically secured in Sylgard-lined dishes as those used for eye motion tracking. During the staining process, the Ringer solution was temporarily removed from the dish to prevent tracer crystals from undesired spreading and contaminating adjacent tissue [31]. Crystals were deposited in the left half of the single-foliated cerebellum. Thereafter, preparations were incubated in 500 ml freshly oxygenated frog Ringer solution at 16 °C for 24 h, fixed in freshly prepared 4% paraformaldehyde (PFA) for 24 h, washed 3 × in phosphate-buffered saline (PBS; 0.3 mM Na_3_PO_4_, 15 mM NaCl, 0.105 mM K_3_PO_4_) for 10 min, counterstained with DAPI (Thermo Scientific, 62,248) at 1:500 for 2 h at room temperature and subsequently cleared using the uDISCO protocol [34]. In short, whole mount preparations were dehydrated for 2 h each in stepwise increasing concentrations of butanol (30, 50, 70, 80, 90, 96, 100%), and cleared for at least 4 h in a 1:2 solution of benzylbenzoate and benzylalcohol, mixed 15:1 with diphenylether. The cleared tissue was mounted in the clearing solution using custom built metal spacers and was scanned on a Leica SP5-2 confocal microscope (LAS-X software) with ~ 400 optical sections of 1.2 µm thickness.

### Immunohistochemistry

Calbindin immunostaining was performed on 10 µm or 30 µm thick parasagittal or coronal brain sections. Following isolation (see above), brains were fixed in freshly prepared 4% PFA for 3 h at 4 °C and were subsequently stored in PBS. Prior to cutting, brains were cryoprotected for at least 48 h in 30% sucrose at 4 °C, and subsequently embedded in tissue freezing medium (Leica, 14,020,108,626) on dry ice, frozen onto a sample holder with the same medium and cut directly onto Superfrost Plus slides (Epredia, J1810AMNZ). Sections were left to dry for 24 h, rehydrated in PBS for 15 min, incubated with a rabbit-anti-Calbindin (Abcam, ab108404) and a mouse-anti-GAD67 antibody (Abcam, ab26116), diluted 1:400 in PBS with 0.05% Triton-X 100 (Roth, 3051.3) and 10% normal goat serum (Millipore, S26) for 18 h at 4 °C. Slides were then washed 3 × for 10 min in PBS and subsequently incubated with an Alexa 488-conjugated goat-anti-rabbit antibody (Invitrogen, A11008), Alexa 546-conjugated goat-anti-mouse antibody (Invitrogen, A21045) and DAPI at a dilution of 1:400, 1:400 and 1:500, respectively, in PBS with 0.05% Triton-X. Slides were then washed 3 × for 10 min in PBS and finally cover-slipped with Poly-Mount (Polysciences, 18,606–20). Sections were scanned on a Leica SP5-2 confocal microscope at an optical thickness of 1.2 µm (10 × objective) or 0.63 µm (20 × objective), with ~ 10 (10x) or ~ 30 (20x) optical sections. For quantification, image stacks were loaded into ImageJ [35] and on each image, the area in µm^2^ of Purkinje cell somata and Calbindin-positive dendritic trees, respectively, was measured. Areas were then multiplied by the thickness of each slice for an approximation of the volume, and summed up across slices for each individual animal.

## Results

Semi-intact preparations of larval *Xenopus* were placed in the center of a circular recording chamber, surrounded by a vertical striped pattern oscillating horizontally at a constant velocity (Fig. 1a). This large-field visual motion pattern elicited eye movements that faithfully followed the stimulus (Fig. 1b), indicative of a robust OKR as demonstrated previously for these animals [27, 29, 36]. Prolonged presentation of this pattern for 30 min, here referred to as optokinetic training, provoked changes in the performance of the OKR. Such OKR modulations were observable in both young (stage 50–51) and old (stage 55–56) *Xenopus* tadpoles, although to different extents in the two groups. Irrespective of age-dependent entrained characteristics, the training evoked plasticity measures with corresponding behaviorally relevant changes.

### Plasticity of optokinetic responses

#### Old tadpoles

**Fig. 2:**
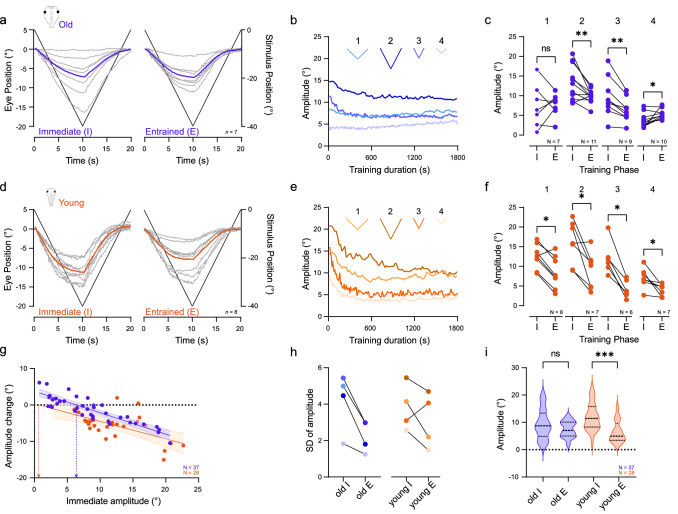
Ontogeny of OKR plasticity.** a–f** Differential effects of prolonged visual image motion in old (**a–c**) and young (**d–f**) larvae; individual eye motion cycles (gray traces) and population average (colored traces, *n* = 7 (old, **a**), *n* = 8 (young, **d**) at the onset (left) and the end (right) of prolonged visual motion stimulation with profile 1 (velocity: 4°/s, cycle duration: 20 s; see 1 in Fig. 1c); population-averaged response amplitudes across 30 min of training (**b, e**), measured on a *per*-cycle basis for stimulus paradigms 1–4, respectively; mean response of five cycles (**c, f**) obtained immediately (I) and at the end of the 30-min OKR entrainment (E) on a *per*-animal basis for stimulus paradigms 1–4 (performed on different, independent sets of naïve tadpoles, respectively; see Fig. 1c). **g** Amplitude changes plotted against immediate OKR amplitudes for old (blue) and young (orange) tadpoles; solid lines indicate linear regression fit to data; shaded areas indicate 95% confidence interval of the fit; horizontal dotted line indicates no amplitude change; dots above indicate OKR amplitude increase, dots below OKR amplitude decrease; blue (old tadpoles) and orange (young tadpoles) vertical dashed arrows indicate the theoretical initial amplitudes, above or below which a decrease or increase is expected. **h** Standard deviation (SD) of the averaged response amplitudes immediately (I) and at the end of the entrainment period (E) for old and young tadpoles and stimulus paradigms 1–4 (for color-code see **b**, **e**). **i** Violin plots of immediate (I) and entrained response amplitudes (E), in young and old tadpoles respectively, pooled across stimulation paradigms 1–4

Optokinetic training with the standard reference motion profile (± 40° positional excursion, ± 4°/s velocity, 20 s period, Stimulus 1; Fig. 1c) consistently elicited changes of the OKR amplitude that varied considerably in extent between individual preparations (Fig. 2a, left). Most tadpoles exhibited an early decrease of the response amplitude within the first 5 min of the training (Fig. 2b), however, the effect of the entrainment on OKR magnitude was rather variable at the end of the 30-min training. While some of the stage 55–56 tadpoles exhibited an amplitude increase or approximately maintained the pre-training amplitude, others showed a considerable attenuation of the OKR by the entrainment (Fig. 2a, right, 1 in Fig. 2c). A detailed evaluation of the emerging pattern suggested that this variability was systematically correlated with the initial amplitude of the OKR prior to the training session (Fig. 2a, 1 in Fig. 2c). Despite the use of the same stimulus (1 in Fig. 1c), the initial, pre-training amplitude differed markedly between individual preparations, with apparently differential consequences for the direction of the training-induced plasticity. While the average response amplitude of the population before (mean ± SD: 7.4° ± 5.4) and after 30 min training (mean ± SD: 7.6° ± 3.0) was very similar (*p* = 0.99; Wilcoxon signed-rank test; 1 in Fig. 2c), the overall variability of the post-training OKR amplitudes became markedly reduced. This effect derived from an overall homogenization of the OKR amplitude of the population after the training. OKR responses with large initial peak-to-peak amplitudes became attenuated during the prolonged visual motion stimulation, while small initial OKR magnitudes became enhanced by the training (1 in Fig. 2c). To test whether the degree and direction of the plasticity indeed depended on the initial response magnitude, motion stimulus parameters that more invariantly elicited either large (2, 3 in Fig. 1c) or small pre-training OKR amplitudes (4 in Fig. 1c) were used.

Presentation of visual motion stimuli with higher velocity and/or excursion magnitudes of the stimulus pattern (2, 3 in Fig. 1c) consistently evoked an OKR with large amplitudes prior to the onset of the training in practically all tadpoles (pattern 2: mean ± SD: 13.4° ± 4.5; pattern 3: mean ± SD: 9.7° ± 5.0; Fig. 2b, c). Prolonged exposure to either of these two visual motion paradigms reliably caused a significant attenuation of the entrained responses (pattern 2: mean ± SD: 9.9° ± 1.8; *p* = 0.0049, Wilcoxon signed-rank test; pattern 3: mean ± SD: 6.4° ± 3.0; *p* = 0.0039, Wilcoxon signed-rank test; Fig. 2c). However, despite reacting to the same stimulus profile, tadpoles with an initial OKR of intermediate amplitude exhibited a weaker attenuation of the entrained OKR amplitudes. We, therefore, suspected that the effective parameter for initiating the robust decrease in OKR amplitude is likely the initial eye motion performance itself, rather than large motion stimulus amplitudes per se. This was tested by presenting a visual motion pattern with smaller stimulus amplitudes (4 in Fig. 1c), which, prior to the training, reliably elicited an OKR with only small response amplitudes (4 in Fig. 2c, mean ± SD: 3.8° ± 1.8). Prolonged exposure to this visual motion stimulus for 30 min robustly provoked a significant increase of the entrained OKR amplitudes (4 in Fig. 2c, mean ± SD: 5.3° ± 1.3; *p* = 0.0137, Wilcoxon signed-rank test).

These experiments demonstrated a response amplitude-dependent plasticity of OKR magnitudes following prolonged exposure to visual motion stimuli in the cohort of old tadpoles. The observed changes comply with the reported cerebellum-dependent homeostatic eye movement plasticity previously demonstrated for the VOR in these animals [26], albeit with a stronger emphasis on motor output magnitude rather than stimulus strength. Based on the arguments presented in the latter study, the currently observed harmonization of the OKR amplitudes towards a preset response magnitude following prolonged visual motion stimulation might also be triggered by a cerebellum-dependent homeostatic plasticity. Within the group of animals with a variety of initial OKR amplitudes, this suggests that with ongoing training, responses become more similar between animals. This interpretation is supported by the clear diminishment of the variability as indicated by the dependency of training-induced changes on initial eye motion amplitudes across different stimulus paradigms, which shows a linear relationship between the initial response amplitude and resultant change by the entrainment (Fig. 2g, blue, *R*^*2*^ = 0.765). The linear regression analysis further allows estimates about the plasticity pattern. The slope of the regression line indicates the dependency of the plasticity on the initial amplitude, with 0 signifying no, and higher/lower values indicating a pronounced dependency. For old, stage 55–56 tadpoles, the slope was -0.59, indicating a clear dependency of the plasticity on the initial OKR amplitude. Furthermore, the x-axis zero-intercept (horizontal dotted line) of the linear regression (blue dashed vertical arrow in Fig. 2g) yielded the theoretical initial amplitude, above or below which a respective decrease or increase is expected. In old tadpoles, this value was 6.4°. Also, the training was accompanied by a reduction of the standard deviation (see above, Fig. 2h), which together with the smaller variation of post-training response amplitudes across the different stimuli (Fig. 2i), indicates an overall reduced inter-animal variability of OKR amplitudes after the training in these animals.

### Young tadpoles

Given the likely influence of the cerebellum in modulating the observed plasticity in visuo-motor behavior, younger tadpoles, with a potentially immature cerebellum, were assessed for similar visuo-motor training-induced changes. Anatomical studies identified the presence of the olivo-cerebellar circuitry in *Xenopus laevis* as early as stage 45 [37, 38]. However the functional onset of all relevant morphological structures occurred only much later at stage 53 [37, 38]. Even though these findings suggest that young tadpoles have defects in the cerebellar control of the OKR, a confirmation by respective behavioral studies is absent. Accordingly, young tadpoles (stages 50–51), which previously have been shown to exhibit a robust OKR [39, 40] were subjected to the same OKR training paradigm as the group of old tadpoles (see above). In general, OKR responses in young tadpoles prior to the training were similarly variable in amplitude for each stimulus paradigm (Fig. 2d, f). The subsequent optokinetic training of young larvae also induced an amplitude decrease early during the prolonged stimulation (Fig. 2e) as in older tadpoles. However, in contrast to the latter group, this decrease became manifested over the 30-min training period such that all young tadpoles exhibited a consistent and significant OKR amplitude reduction after the entrainment (*p* < 0.05 for 1–4 in Fig. 2f; Wilcoxon signed-rank test) independent of the stimulus profile and thus of the initial response magnitude (compare “I” and “E” in Fig. 2f).

Furthermore, and also in contrast to the outcome reported for older tadpoles, the training-induced diminishment of the OKR response within this younger age-group was not accompanied by a strong, concurrent reduction in the variability of the respective peak-to-peak amplitudes as observed in older animals (Fig. 2g). This is illustrated by the retention of the relatively large variability of entrained response amplitudes in young tadpoles (orange dots in Fig. 2g; *R*^*2*^ = 0.378) as compared to the group of older tadpoles (blue dots in Fig. 2g; *R*^*2*^ = 0.765). This notion was substantiated by the rather limited reduction of the standard deviation of the entrained as compared to the immediate response amplitudes (Fig. 2h) and by the reduced distribution of post-training amplitudes across all stimuli (Fig. 2i). While initial peak-to-peak responses in young animals appeared to be larger in magnitude (compare Fig. 2a and d), the effect was not significant across paradigms (compare young “I” and old “I” in Fig. 2i). Nonetheless this finding might have been influenced by the overall larger peak-to-peak amplitudes of the OKR for the smallest stimulus, specifically at young stages, as compared to older tadpoles (Supplemental Fig. 1a, stimulus profile 4, *p* = 0.0185, Mann–Whitney *U*-test). While this fact does not explain the absent homogenization of inter-animal variations after the entrainment in young tadpoles, this aspect might well contribute to the lack of amplitude increases. This effect might emerge because even the smallest stimulus (4 in Fig. 1c) triggered OKR responses in younger larvae with amplitudes that generally exceeded those encountered in older tadpoles and which were increased after training in the latter (see vertical dashed line indicating the transition from amplitude increases (above) to decreases (below) in Fig. 2g). However, an estimate can be made based on the linear regression analysis shown in Fig. 2g. The slope of the regression line in young larvae (– 0.48) was lower than that of old tadpoles, indicating a rather limited dependency of the direction and strength of the plasticity on the initial amplitude of stage 50–51 larvae. In addition, the x-axis zero-intercept (horizontal dotted line) at 0.6° (orange dashed vertical arrow in Fig. 2g) indicates that much smaller amplitudes are theoretically required for an amplitude increase. The lack of amplitude increases by the entrainment in young tadpoles and the initially larger OKR amplitudes in these animals might derive from immature cerebellar circuits at this developmental stage [38], with a consequent lack of modifiable inhibitory influences of Purkinje cells on, e.g., vestibulo-ocular neurons and thus on the activation of extraocular motoneurons [6]. To test and eventually confirm the role of the cerebellum in OKR plasticity in *Xenopus* tadpoles, the neuronal connection between inferior olivary neurons and the cerebellum, which would serve as a critical pathway for such a computation [10], was next surgically interrupted in old tadpoles.

### Impact of inferior olivary projections on ocular motor plasticity

**Fig. 3:**
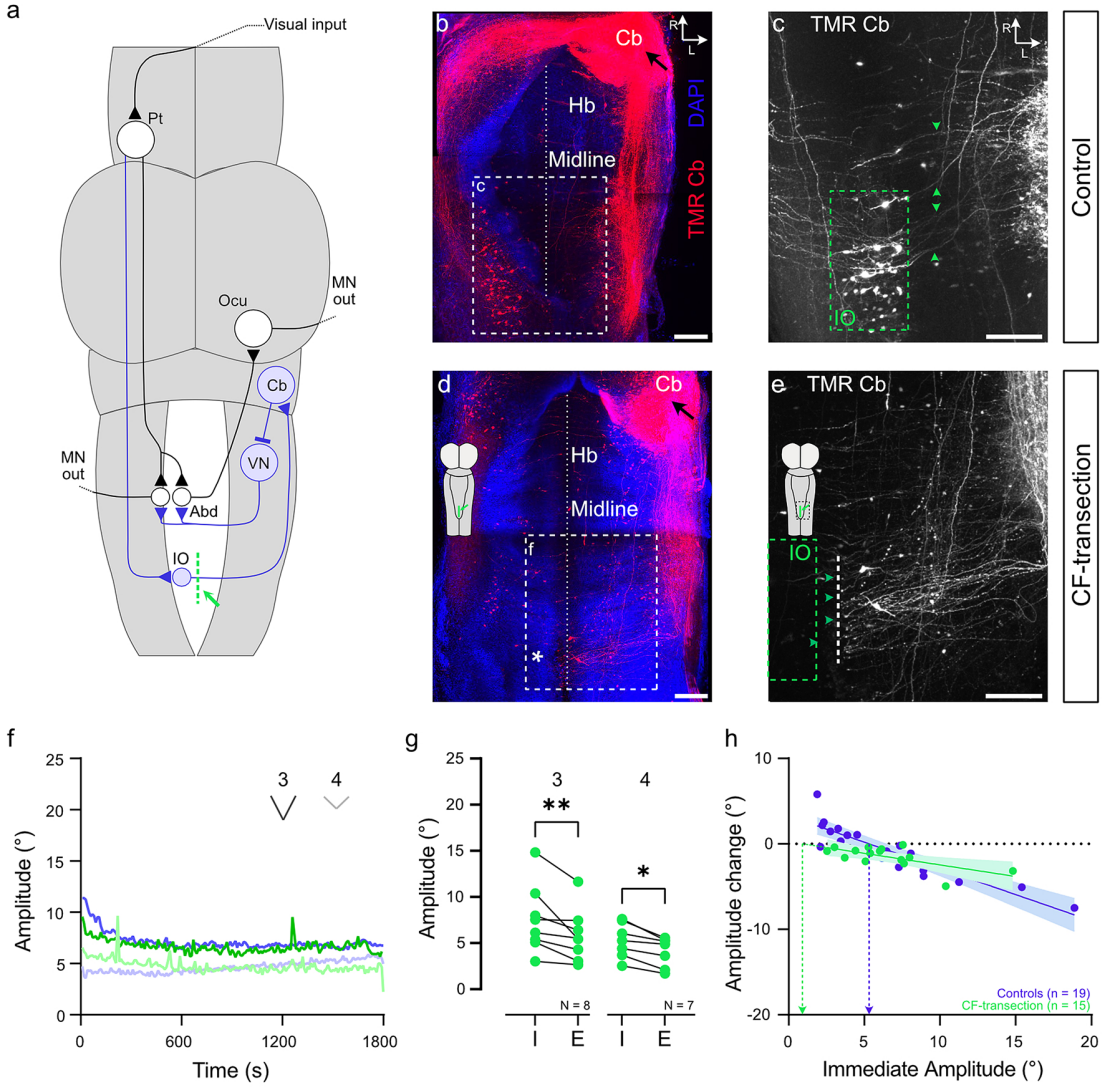
Anatomical and functional consequences of climbing fiber transection.** a** Schematic of a *Xenopus* tadpole brain depicting the direct (black) and indirect (blue) OKR pathways for eliciting a horizontal OKR during rightward motion stimulation of the right eye; the site of climbing fiber transection in the caudal hindbrain is indicated by the green dashed line. **b**–**e** Whole-mount confocal reconstructions of the hindbrain (Hb) of a stage 55 control tadpole (**b–c**) and after midline transection (**d–e**) at the level of the caudal hindbrain (green line in **a**, scheme in **d**) counterstained with DAPI (blue nuclei); unilateral cerebellar injections of Tetramethylrhodamine (TMR; black arrows), outlined the climbing fiber (CF) axonal pathway (green arrow heads in **c, e**) and parent inferior olivary cell bodies in the contralateral ventral hindbrain in controls (**b**) and lack thereof after the lesion (* in **d**), illustrated at higher magnification in **c, e** (green dashed rectangles); **f** Population-averaged response amplitudes across 30 min of training, measured on a *per*-cycle basis for stimulus paradigms 3 and 4, in controls (blue) and after CF transection (green). **g** Mean response of five cycles obtained immediately (I) and at the end of the 30-min OKR entrainment (E) on a *per*-animal basis for stimulus paradigms 3 and 4 (see Fig. 1c) in CF-transected animals. **h** Amplitude changes plotted against immediate OKR amplitudes for controls (blue) and CF-transected animals (green); solid lines indicate linear regression fit to data, and shaded area the 95% confidence interval of the fit; horizontal dotted line indicates no amplitude change; dots above the line indicate amplitude increase, dots below amplitude decrease. Blue (control tadpoles) and green (CF-transected tadpoles) vertical dashed arrows indicate the theoretical initial amplitudes, above or below which a decrease or increase is expected. *Abd* abducens nucleus, *Cb* cerebellum, *D* dorsal, *IO* inferior olive, *L* lateral, *MN* motoneurons, *Ocu* oculomotor nucleus, *Pt* pretectum, R rostral, *VN* vestibular nuclei. Scale bars in all panels represent 100 µm

The climbing fiber input to the cerebellum (Fig. 3a) arising from the inferior olive (IO) (Fig. 3a–c) was surgically interupted by a midline transection at the level of the caudal hindbrain in the group of old tadpoles (Fig. 3a, green line; d, e, inset). This manipulation was preferable over a pharmacological or surgical inactivation of the cerebellum itself, because transection of the climbing fiber pathway specifically eliminates visual reafferent signals required for the execution of plastic alterations, while leaving other neuronal elements of the cerebellum, such as parallel fiber inputs, and their effect on the OKR anatomically and functionally intact. Dye labeling of these projections was used to confirm the integrity or interruption of the midline-crossing axonal pathway ventrally in the caudal hindbrain (Fig. 3c green arrowheads). Labeled fibers in controls represent axons of inferior olivary neurons (Fig. 3c, green rectangle). Following midline transection, these axons were disconnected from their parent cell bodies (Fig. 3d, *), as indicated by the failure to retrogradely label the latter somata from the cerebellum (Fig. 3e, green square). These results anatomically confirmed the efficacy and success of the approach in functionally disconnecting the cerebellum from the inferior olivary nucleus. Following transection of the climbing fiber pathway, respective preparations were subjected to prolonged OKR training as described above with a stimulus pattern that either elicited large (3 in Fig. 1c; 3f; mean ± SD: 7.5° ± 3.7) or small (4 in Fig. 1c; 3f; mean ± SD: 5.3° ± 1.8) peak-to-peak response amplitudes. The magnitude of the initial OKR after the surgery was comparable to those of controls (Supplemental Fig. 1b, *p* = 0.2359 for 3; *p* = 0.1088 for 4, Mann–Whitney *U*-test), suggesting that the lesion had no significant impact on the general performance of the OKR. The retention of pre-lesion OKR amplitudes likely derived from the employed lesion protocol that left cerebellar circuits anatomically unimpaired. In addition, a reversal of potential long-lasting, downstream manifestation of previously acquired cerebellar influences on the OKR circuitry [41, 42] was avoided by the brevity of the experiments. In addition, prolonged visual motion stimulation consistently provoked an OKR amplitude decrease in all preparations during the early phase of the training (Fig. 3f, green traces). However, in contrast to old control tadpoles with intact climbing fibers, animals with a climbing fiber transection only expressed a consistent and significant down-regulation of the OKR amplitude after the 30-min training, irrespective of the initial amplitude (3 in Fig. 3g: mean ± SD: 7.5° ± 2.6, *p* = 0.0078; 4 in Fig. 3g: mean ± SD: 4.1 ± 1.6, *p* = 0.0156; Wilcoxon signed-rank test). This result was not surprising for tadpoles with large initial amplitudes prior to the training as shown in the age-matched controls (Fig. 3f, top green and blue traces, 3 g, left). However, that this decrease was observed in animals subjected to stimuli which normally provoke a robust OKR increase over time was in stark contrast to controls (Fig. 3f, bottom green and blue traces). The rather invariant and unidirectional training effect, irrespective of the initial amplitude (Fig. 3g, h), resembled the exclusive attenuation of the OKR amplitude by prolonged visual motion stimulation in young larvae (Fig. 2f, g), with an even lower slope of the regression line of -0.29 and a comparable x-axis zero-intercept (horizontal dotted line) at 0.94 (green vertical dashed arrow in Fig. 3h). This suggests that the climbing fiber input to the cerebellum is involved in an up-, but not in a down-regulation of the OKR amplitude. Accordingly, the lack of an amplitude increase of the OKR at young larval stages might derive from the fact that the cerebellar circuitry and/or respective cellular elements are incomplete or insufficiently mature to exert a homeostatic increase of the ocular motor output.

### Ontogeny of cerebellar elements

**Fig. 4:**
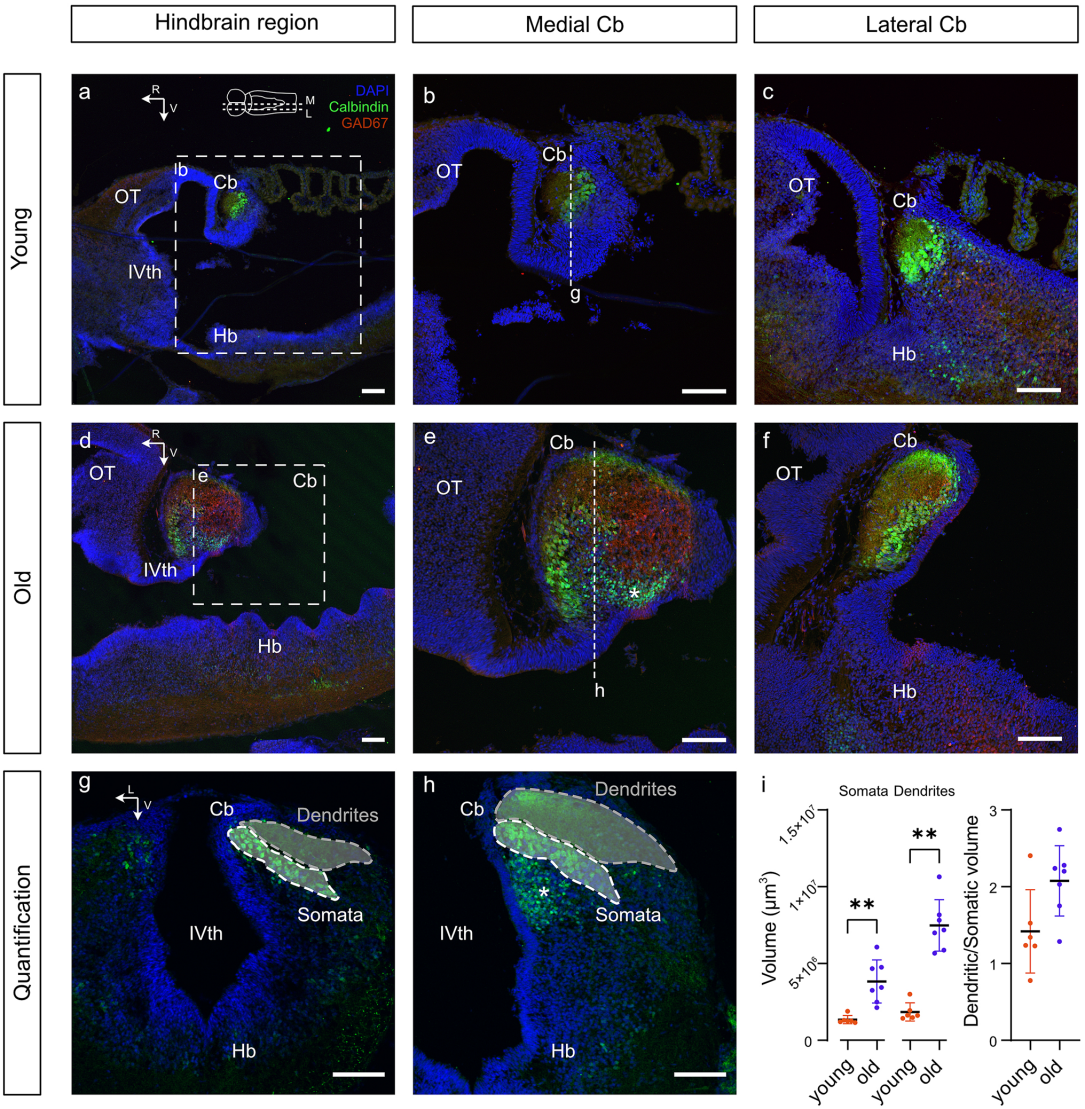
Ontogenetic plasticity of calbindin- and glutamate-decarboxylase (GAD67)-immunopositive cerebellar structures.** a–f** Parasagittal sections at two medio-lateral planes (see scheme on top in **a**) through the hindbrain (Hb) depicting calbindin- (green) and GAD67-labeled (red) and DAPI-counterstained (blue) morphological structures in young (**a–c**) and old tadpoles (**d–f**); medially (M in scheme on top in **a**) located sagittal sections depicting overviews (**a, d**) and higher magnifications (**b, e**) of calbindin- and GAD67-immunopositive structures in the hindbrain and cerebellum (Cb); laterally (L in scheme on top in **a**) located sagittal sections (**c, f**) depicting respective immunopositive elements in the lateral cerebellum of young (**c**) and old (**f**) tadpoles. **g, h** Coronal sections at the level of the cerebellum of young (**g**) and old (**h**) tadpoles, outlining the region used to measure the location of Purkinje cell (PC) somata (white) and the extension of the dendritic tree (gray). **i** Quantification of the volumes of Purkinje cell somata (left), dendritic tree (middle) and ratio of dendritic tree/Purkinje cell somatic volumes (right) in young and old tadpoles. *OT* optic tectum, *R* rostral, *V* ventral, *IVth* fourth ventricle. Scale bars in all panels represent 100 µm

To explore whether developmental differences at the level of the cerebellum might explain the observed differential OKR plasticity in younger as compared to older tadpoles, immunohistochemical analyses of key cellular elements at stage 50 and stage 56 were performed (Fig. 4). These developmental stages coincided with the condition prior to and after the known onset of Purkinje cell and climbing fiber development in frogs [43]. The calcium-binding protein Calbindin reliably outlined Purkinje cell somata and dendrites at both developmental stages and allowed a comparison of the general cerebellar anatomy and cell morphology (Fig. 4). Apart from the expected size difference of the cerebellum due to body and brain growth between younger and older tadpoles (Fig. 4a, d), the cerebellum at stage 56 revealed a more elaborate structural diversification (Fig. 4b, e). Combined with immunohistochemical labeling for GAD67, a marker for GABAergic neurons, younger tadpoles revealed the characteristic Purkinje cell layer and a molecular layer. The corresponding structural elements at stage 56, in contrast, had a very different and considerably layered organization of these zones (Fig. 4e), in addition to another group of Calbindin-positive cells that were smaller in size when compared to Purkinje cells (* in Fig. 4e). In comparison to young tadpoles, the molecular layer at stage 56 was expanded and encompassed a large region with GABAergic cell bodies and fibers and a predominantly Calbindin-immunopositive dorsal ridge. In addition, while cerebellar structures at stage 50 were rather homogenous in the medio-lateral direction (Fig. 4b, c, g), the molecular layer, and in particular the GABAergic components, increased in extent towards the midline of the cerebellum (Fig. 4e, f, h). Quantification of the volume of both the Purkinje cell somata and dendrites revealed a significant increase for both parameters during development. The approximate Purkinje cell layer volume increased significantly from 1.3 × 10^6^ µm^3^ ± 0.2 × 10^6^ (mean ± SD) in young to 3.8 × 10^6^ µm^3^ ± 1.4 × 10^6^ (mean ± SD) in old tadpoles (Fig. 4i, Somata, *p* = 0.012, Mann–Whitney *U*-test). The approximate dendritic volume also markedly increased from 1.8 × 10^6^ µm^3^ ± 0.6 × 10^6^ (mean ± *SD*) in young to 7.4 × 10^6^ µm^3^ ± 1.6 × 10^6^ (mean ± SD*)* in old tadpoles (Fig. 4i, Dendrites, *p* = 0.012, Mann–Whitney *U*-test). While general increases in volume were largely due to the brain growth during development, the overall Purkinje cell dendritic volume appeared to increase disproportionately compared to the layer of Purkinje cell somata. While not quite significant (*p* = 0.07), the dendritic volume was 2.1 ± 0.5 (mean ± SD) times larger than the area occupied by the cell bodies in old tadpoles, while this ratio was only 1.4 ± 0.5 (mean ± SD) in young tadpoles (Fig. 4i). Therefore, in addition to the lack of a layered population of small Calbindin-positive cells, those layers, which were already present in the cerebellum of young tadpoles are considerably smaller compared to those in older animals. As climbing fiber–Purkinje cell interactions conveying visual sensory feedback to the cerebellum occur in the molecular layer at Purkinje cell dendrites, the anatomical findings provide suggestive evidence for a potential substrate of the observed developmental differences in OKR plasticity.

## Discussion

The OKR in *Xenopus* tadpoles at mid-larval stages exhibits a bidirectional plasticity during prolonged visual motion stimulation. The outcome of the entrainment was rather variable, and in different animals consisted of an increase, a decrease, or no alteration of OKR magnitudes. The overall variable gain changes, however, were inversely correlated with the pre-training response amplitude, suggesting the activation of a homeostatic plasticity that likely adjusts the motor performance to a preset value during prolonged stimulation. In contrast to this bidirectional plasticity of older tadpoles, younger larvae exclusively exhibited a decrease of the OKR amplitude, suggesting the presence of two separate plasticity mechanisms with different ontogenetic onsets. The neuronal site for the delayed up-regulatory plasticity likely includes inferior olivary–cerebellar projections and requires functionally mature cerebellar elements, suggested by respective morphological differences between young and old tadpoles.

### Substrate and development of OKR performance

The sensory–motor transformation subserving the OKR occurs along two different neuronal pathways (Fig. 3a) [3]. In both circuits, information about visual image motion is mediated by sets of retinal ganglion cells in the accessory optic tract that terminate in several, motion direction-specific, nuclei in the pretectum [44]. These pretectal nuclei represent the hub for a direct projection to extraocular motoneurons as demonstrated, e.g., in frogs, forming a short-latency OKR circuitry (Fig. 3a, black) [45, 46]. A second, more indirect pathway encompasses projections from the pretectal area through the inferior olive, cerebellum, and vestibular nuclei to finally access motoneurons in the extraocular motor nuclei (Fig. 3a, blue) [3]. This longer-latency pathway, involving the cerebellum, allows integration of multimodal signals related to motion in space and provides the likely substrate for spatio-temporal plasticity of ocular motor reactions in all vertebrates [47] including amphibians [26]. This pathway is shared with the VOR, through the projection of Purkinje cells onto floccular target neurons in the mammalian vestibular nuclei [12] or the correspondent cell type in amphibians to modulate the VOR gain through direct projections onto ocular motor nuclei (Fig. 3a). In fact, visual feedback in the form of climbing fiber activity onto Purkinje cells is essential for VOR adaptation [42, 48], linking OKR and VOR plasticity. Thus, visuo- and vestibulo-motor plasticity are at least in part governed by shared substrates and neuronal principles, indicating that the mechanistic features, such as spatio-temporal as well as eco-physiological circumstances under which behavioral adaptations are induced might be similar and likely cross-linked between the visuo- and vestibulo-motor system, once the cerebellum has become functional.

As precocial animals, locomotor activity in *Xenopus laevis* emerges very soon after hatching [49], which necessitates concurrent gaze-stabilizing eye movements [50]. While this is achieved at very early developmental stages by feedforward locomotor efference copies and utricular signals [40, 51, 52], the OKR is required to provide visual feedback and to initiate modifications of eye movements [52–54]. In fact, a functional OKR has been observed in *Xenopus* tadpoles as early as stage 45, with a moderate performance that remains largely invariant across the subsequent developmental period [51, 52]. These visuo-motor responses are thus well suited to provide appropriate visual feedback [55] to fine-tune gaze-stabilizing eye movements evoked by locomotor efference copies and/or vestibulo-ocular reflexes [40, 51, 52] at both developmental stages of *Xenopus* larvae employed in the current study.

Despite the presence of a robust OKR, the age-dependent differences in the capacity to exert plastic adaptations of the performance indicate that part of the sensory–motor circuitries or individual cellular elements might still be incomplete or afunctional at early larval stages. The robust performance, though without the ability for bidirectional changes at early developmental stages, suggests that visuo-motor responses in young larvae are exclusively mediated by the direct pathway (Fig. 3a, black). Given that the cerebellum is the predominant site for initiating modifications of eye movements in all vertebrates [10] including amphibians [26], it is possible that the indirect visuo-motor pathway through the cerebellum is still insufficiently established in young larvae, in compliance with the morphological findings of the current study (Fig. 4). This interpretation depends on the assumption that the rather stereotypic down-regulation of OKR performance across animals and stimuli can occur independent of intact climbing fiber connections as demonstrated by the respective lesion (Fig. 3), and may be caused by habituation or fatigue. While the overall consequence on OKR amplitude is similar between lesioned old and un-lesioned young animals, the underlying mechanism of the down-regulation most likely differs from the amplitude decreases previously observed during long-term visuo-vestibular mismatch training, based on the considerably slower time-course of the latter experimental outcome and the multiple timescales and mechanisms identified for motor learning [16, 56, 57].

### Ocular motor plasticity

While young *Xenopus* tadpoles prior to stage 51 exhibit already some degree of visuo-motor plasticity, the full range of OKR adaptability appears only later during ontogeny. The OKR magnitude-dependent bidirectional adaptation caused an overall harmonization of the response amplitudes across different animals as well as across different stimuli. As such, these data suggest a rather more homogenous entrained performance, indicative of a homeostatic mechanism. Prerequisite to this homeostatic plasticity is the variability of the naïve OKR before training. A comparably variable inter-individual performance has been observed for the goldfish [58] and *Xenopus* tadpole OKR [55], as well as for the *Xenopus* VOR [59], and is likely related to intrinsic sensory–motor noise levels in the system as indicated in the latter study. This also includes variations in retinal sensitivity to the stimulus, as well as different muscle strengths and differences in the activity levels in the underlying neuronal circuits. Furthermore, the conduction of the experiments in developing brains likely enhances these effects, potentially inducing a larger degree of output variability. The displayed adaptive plasticity likely aims at maintaining a defined and preset level of synaptic drive within the visuo-motor circuitry during continuous activation. However, such an adaptation, specifically the attenuating component, is in contrast to those previously observed in fish [19] or mammals [53, 60], where prolonged OKR training consistently induced an increase of the OKR response, ultimately leading to a higher and thus more efficient visual motion tracking. This difference might derive from differences in stimulus conditions or might represent a species-specific feature. The bidirectional changes in older *Xenopus* tadpoles suggest that the homogenization of OKR amplitudes through training aims at an optimization of motor performance rather than an enhancement of tracking a large-field visual scene. This concept of an adaptive plasticity, i.e., dynamic context-dependent increase or decrease of eye movements to achieve a leveled response, has previously been demonstrated for the VOR in age-matched *Xenopus* tadpoles [26]. For the respective training paradigm in the latter study, the direction of VOR amplitude changes were governed by stimulus, rather than response strength as demonstrated here. This difference complies with the fact that the OKR is a closed-loop system, operating on sensory feedback, allowing a graded increase or decrease of the amplitude rather than a stereotypic enhancement or attenuation. The cellular substrate for such differentially tuned plasticity is located in the molecular layer of the cerebellum (see [8, 15, 61]), and at least partially conveyed by climbing fiber input from the inferior olive, which has been shown to correlate with an increase, but not a decrease of VOR gain [62, 63]. As two of several cerebellar learning mechanisms, long-term potentiation (LTP) of parallel fiber–Purkinje cell synapses, diminishes the performance of gaze-stabilizing reflexes, while long-term depression (LTD) of such synapses, induced by climbing fiber and Purkinje cell co-activation, increases reflex performance [64, 65]. In both cases, this is achieved by modulating Purkinje cell firing rates, which by itself is sufficient to decrease or increase VOR performance [66]. This increase *versus* decrease is supplemented by the modulatory activity of GABAergic molecular layer interneurons, known to fine-tune the impact of climbing fiber activity for more nuanced gain changes, potentially facilitating adaptive plasticity as observed in the current study [67]. A comparable role of GABAergic neurons in the molecular layer of the cerebellum in *Xenopus* occurs likely in old, but not young tadpoles (Fig. 4). The fact that young *Xenopus* larvae lack a cerebellar granule cell layer [38], required for LTP-induced response decreases, supports the notion that the observed amplitude decrease at early developmental stages might be conveyed by a non-cerebellar pathway, although this remains to be experimentally validated.

### Development of cerebellar circuits as anatomical substrate for ocular motor plasticity in *Xenopus*

The differences between the two larval age-groups correlate with known checkpoints in cerebellar development, and the ontogenetic appearance of cerebellar structures in this species, further tying behavioral differences to this brain structure. While inferior olivary–cerebellar connections are present as early as stage 45, climbing fiber axons are sparse, unlikely to be functional, and Purkinje cell dendritic trees are only rudimentary in extent [37]. In addition, the external granule cell layer in the *Xenopus* cerebellum remains inconspicuous for a substantial part of the larval life and becomes identifiable only at stage 53 [37]. The involvement of cerebellar granule cells in OKR plasticity [68] renders these neurons in particular a possible substrate for the observed training effect. In fact, the immunohistochemical identification of selected cerebellar markers demonstrated rather negligible numbers of major cellular elements at stage 51 (Fig. 4), in compliance with the relatively delayed onset of cerebellar function in comparison to the principal visuo-motor performance (Fig. 2) [52]. Most importantly, however, the volume of Purkinje cell dendrites, the primary site of climbing fiber–Purkinje cell interactions associated with eye motion plasticity [42, 69, 70], is much less pronounced in such young tadpoles as compared to those at stage 56. The rather immature cerebellum at stage 51 likely renders this structure incapable of appropriate, sensory feedback-based neuronal computations to alter visuo-motor responses on a meaningful level. Confirmation for the necessary integrity of the inferior olive and climbing fiber connectivity with Purkinje cells as potential substrates for OKR modifications derives from studies in mice [18]. Based on the outcome of the latter study, pharmacological inactivation of the inferior olive completely eliminates a successful OKR gain-up training, in agreement with the outcome of the transection of climbing fibers in the present study on *Xenopus* tadpoles. According to the collectivity of findings and assumptions therefore, plastic increases of eye movement amplitudes also in *Xenopus,* appear to require a functionally intact inferior olive and ascending connections with cerebellar Purkinje cells, which in *Xenopus* tadpoles reach functional maturity only between stages 51 and 55. Thus, during larval development, a fast onset of the OKR to facilitate gaze stabilization during locomotion from early on in development is only later complemented by a second, plastic pathway recruitment that allows more mature animals to integrate and modify visual feedback. This renders the visual system able to rapidly respond to immediate visual scene motion, within a motor range that is tuned to the animal’s visual surroundings through means of homeostatic plasticity.

## Supplementary Information

Below is the link to the electronic supplementary material.

Supplemental fig. 1 Comparison of OKR amplitudes at training onset (immediate). a Violin plots of immediate OKR amplitudes (prior to the training) in old (blue; n = 37) and young (orange; n = 28) control tadpoles for the different stimulation paradigms (1–4) depicted in Fig. 1c. b Violin plots of immediate OKR amplitudes (prior to the training) in old unmanipulated (blue; n = 19) and climbing fiber (CF)-transected tadpoles (green; n = 15) for stimulus paradigms 3 and 4. * p < 0.05, Mann–Whitney U-test
