# Supplementary figures and images for: Homeostatic plasticity of eye movement performance in Xenopus tadpoles following prolonged visual image motion stimulation
Source: J Neurol. 2022 Aug 10;270(1):57–70. doi: 10.1007/s00415-022-11311-8 (PMC9813097; doi:10.1007/s00415-022-11311-8)

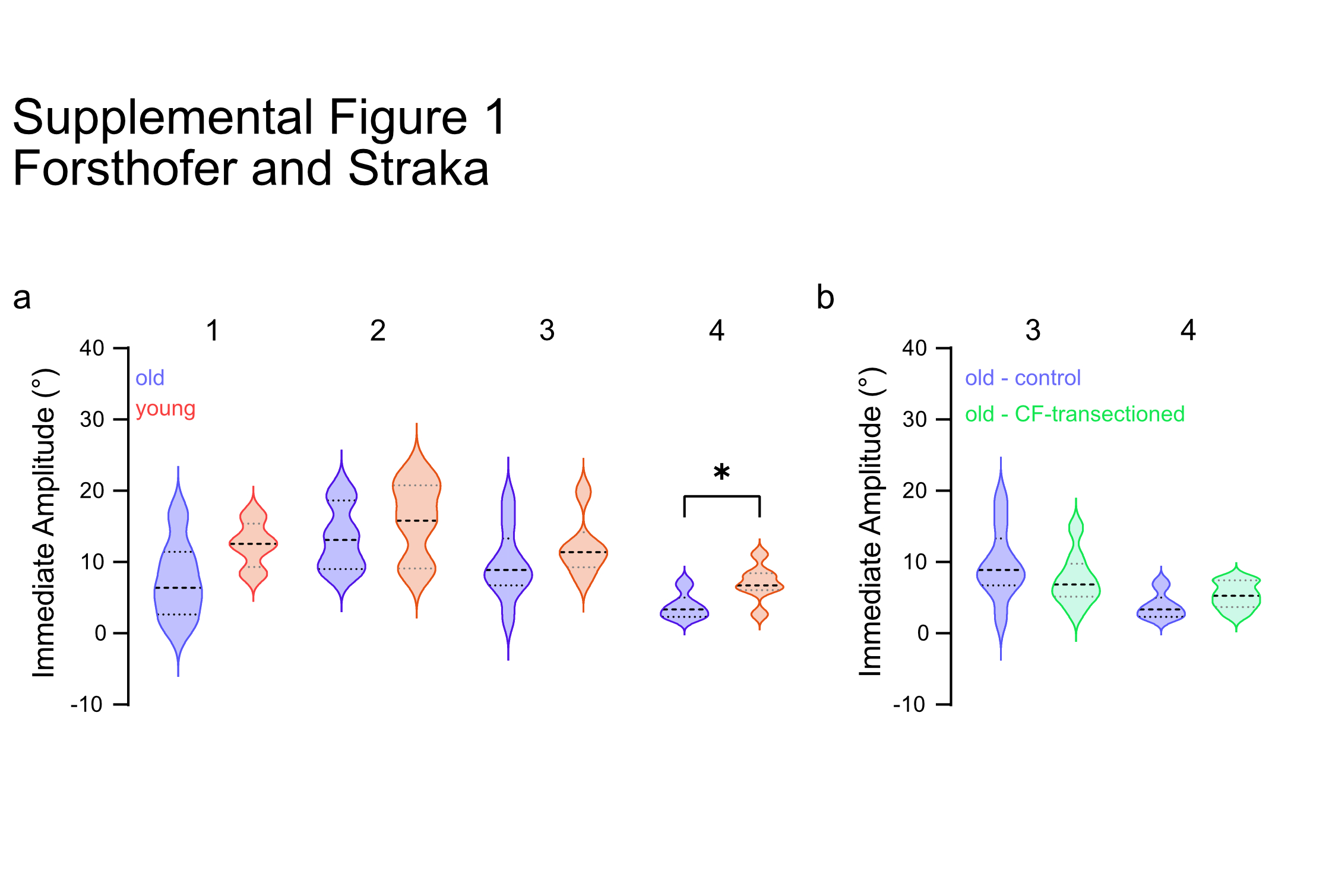

Supplement: Supplementary file 1 — Supplemental fig. 1 Comparison of OKR amplitudes at training onset (immediate). a Violin plots of immediate OKR amplitudes (prior to the training) in old (blue; n = 37) and young (orange; n = 28) control tadpoles for the different stimulation paradigms (1–4) depicted in Fig. 1c. b Violin plots of immediate OKR amplitudes (prior to the training) in old unmanipulated (blue; n = 19) and climbing fiber (CF)-transected tadpoles (green; n = 15) for stimulus paradigms 3 and 4. * p < 0.05, Mann–Whitney U-test [file 415_2022_11311_MOESM1_ESM.jpg]
